# The Dynamics of Cardiovascular Risk—An Analysis of the Prospective Urban Rural Epidemiology (PURE) Poland Cohort Study

**DOI:** 10.3390/jcm13133728

**Published:** 2024-06-26

**Authors:** Paweł Lubieniecki, Łukasz Lewandowski, Maria Wołyniec, Katarzyna Połtyn-Zaradna, Katarzyna Zatońska, Andrzej Szuba

**Affiliations:** 1 Clinical Department of Diabetology and Internal Disease, Wroclaw Medical University, Borowska Street 213, 50-556 Wroclaw, Poland; 2 Department of Medical Biochemistry, Wroclaw Medical University, Chalubinskiego 10, 50-368 Wroclaw, Poland; lukasz.lewandowski@umw.edu.pl; 3 Population Health Department, Wroclaw Medical University, 50-345 Wrocław, Poland; maria.wolyniec@umw.edu.pl (M.W.); katarzyna.poltyn-zaradna@umw.edu.pl (K.P.-Z.); katarzyna.zatonska@umw.edu.pl (K.Z.); 4 Calisia University, 62-800 Kalisz, Poland; 5 Clinical Department of Angiology and Internal Disease, Wroclaw Medical University, Borowska Street 213, 50-556 Wroclaw, Poland; andrzej.szuba@umw.edu.pl

**Keywords:** epidemiological study, cardiovascular risk, lipid–lowering therapy

## Abstract

**Background**: The purpose of this study was to analyze the major cardiovascular risk (CVR) factors and their trends in the study population. **Methods**: The results of subjects in the Polish Prospective Urban and Rural Epidemiological Study (PURE) study group were interpreted. CVR was calculated for each participant according to the Systematic Coronary Risk Evaluation (SCORE2) scale or the Systematic Coronary Risk Evaluation–Older Persons (SCORE2–OP) scale. Data from the beginning of the analysis (2013) and nine years later (2022) were included. In addition, the use of lipid–lowering therapy (LLT) and meeting the low–density lipoprotein cholesterol (LDL–c) target criterion at the beginning and end of the study were analyzed. **Results**: Patients in the high and very high CVR groups who had abnormal LDL–c results accounted for 64% and 91% of their group in 2013 and 70% and 92% in 2022, respectively. **Conclusions**: Regardless of age, patients using LLT at the start of the analysis had a greater increase in future CVR, especially if they had lipid abnormalities at the start of the study. This may be due to reverse causality and multimorbidity in these patients, highlighting the importance of appropriate treatment of lipid abnormalities.

## 1. Introduction

Data from the latest World Heart Federation (WHF) report show that the number of deaths worldwide from cardiovascular causes increased from 12.1 million in 1990 to 20.5 million in 2021. A total of 80% of the deaths occurred in low– and middle–income countries (LMICs) [1]. In Poland, classified as a high–income country, the Polish Society of Cardiology estimates that cardiovascular disease (CVD) accounts for 37% of all deaths. Given the constant progress and developments in various aspects of medicine, such as improvements in endovascular techniques, imaging, or the introduction of new drugs and the optimization of treatment regimens for CVD and comorbidities, these figures are particularly worrying.

In line with the Hippocratic maxim “prevention is better than cure”, as a society, we should place greater emphasis on primary prevention, that is, by controlling potential risk factors, and preventing disease before it occurs. Such non–specific primary prevention of CVD includes education, which should be the cornerstone of any healthy society. Even the smallest lifestyle interventions can be fundamental to quality of life and reduction of major adverse cardiovascular events (MACE). These modifiable aspects include regular physical activity, a diet based on healthy eating principles and possible weight reduction, smoking cessation, and stress reduction.

Other modifiable risk factors are lipid disorders and abnormal blood pressure measurements. According to data from the National Health Fund, hypercholesterolemia is present in almost 60% of the adult Polish population and hypertension has been diagnosed in almost 10 million Poles [2]. The WOBASZ II study described a situation in which 60% of the study group with hypercholesterolemia were unaware of their current lipid disorders, and only 6% of participants had properly treated lipid management [3]. Similarly, lack of knowledge in the population characterizes the second modifiable risk factor, hypertension. Nowicki et al. described a situation in which only 55.33% of respondents identified hypertension as a cardiovascular risk factor (CVR) [4].

Two of the latest tools for predicting cardiovascular events are the SCORE2 (Systematic Coronary Risk Estimation2) scale and the SCORE2–OP (Systematic Coronary Risk Estimation2–Older Persons) scale for people without diagnosed atherosclerotic cardiovascular disease (ASCVD), chronic kidney disease (CKD), or diabetes mellitus (DM). The resulting score is a measure of the 10–year risk of CVD (heart attack, stroke) leading or not leading to death. The risk calculation takes into account smoking, gender, age, non–high–density lipoprotein–cholesterol fraction (nHDL–c), and systolic blood pressure (SBP). For people with CVD, DM, or CKD, the risk is determined according to current guidelines [5,6], taking into account the duration of the disease, laboratory parameters, or the presence of complications.

These models, and their results, can be used to inform patients about potential risks, the benefits of lifestyle modification, or the initiation of appropriate treatment. They can facilitate shared strategic decision–making between clinicians and patients regarding CVD risk management.

The aim of the study was to analyze trends in individual risk factors, achievement of lipid control goals and blood pressure measurements, and use of antihypertensive (AT) and lipid–lowering treatment (LLT) in the entire study cohort, as well as in individual CVR groups. The results are presented below.

## 2. Materials and Methods

### 2.1. Study Group

The analyzed group consisted of participants in the Polish part of the Prospective Urban and Rural Epidemiological (PURE) study residing in Wroclaw and surrounding rural areas. Each participant gave written consent to participate in long–term follow–up with periodic health monitoring. Data were collected during direct contact with each participant in cycles of every 3 years, starting in 2007. Information from 2013 and 2022 on patients without ASCVD, CKD, and DM was included in this study, as it was in these editions that all data necessary for the relevant analyses were available. The Bioethics Committee at the Medical University of Wroclaw gave a positive opinion on the conduct of the study in 2007 (No. KB–443/2006).

The survey instrument consisted of individual questionnaires on lifestyle, chronic diseases, or pharmacotherapy used, as well as home questionnaires. In addition, blood pressure was measured twice in each participant and anthropometric examinations including measurements of height, weight, and waist and hip circumference were performed. Venous blood was also collected, in which lipid profile and renal function parameters were determined. Participants over the age of 35 who gave informed verbal and written consent were included in the study, according to the guidelines of the PURE study. There was no exclusion criterion [7,8].

In this study, we used the SCORE2 and SCORE2–OP scales for a high–risk country. Poland was assigned to this category based on national CVD mortality rates published by WHO [9]. Then, based on age, sex, smoking, nHDL–c levels, and systolic blood pressure, an individual CVR was calculated for each patient, and the participant was assigned to one of four categories—low, moderate, high, or very high risk according to the SCORE2 and SCORE2–OP tables—appropriate for value and age [10,11].

The analysis in this study did not include individuals with significant cardiovascular disease, renal disease, or carbohydrate disorders. These individuals were assigned to the high or very high CVR category based on relevant guidelines [5].

### 2.2. Statistical Analyses

Data preprocessing, statistical modelling, and visualization were performed in STATISTICA 13.3 on the license of Wroclaw Medical University. Multi–way repeated measures ANOVA was performed to explore the multi–factor–adjusted (marginal) effect of factors (effects) taken into account in this study on the time–wise change (2013 vs. 2022, denoted as “TIME” in the manuscript) in CVR. This analysis was performed on the whole set of 2nd–degree interactions between “TIME” and each effect, in one model.

Subsequently, higher–degree effects were explored to analyze the influence of treatment– and demographical–related effects on treatment–wise changes in the time–related dynamics of the CVR. For this purpose, 3rd– or (eventually) 4th–order interactions were analyzed in the process of derivation of separate models and expanding them with new features. *p* < 0.05 was considered significant.

## 3. Results

### 3.1. Overall Outcomes of the Study Cohort and Lipid Disorder Scores in Each CVR Group

In 2013, the population sample counted 1153 participants. These were individuals who had a set of necessary data and a CVR group could be calculated for them. In 2022, 9 years later, the group represented 995 people. A total of 113 people refused to participate further in the study and 45 people died. The low CVR group in 2013 comprised 333 people. A total of 31% of this group had uncompensated lipid disorders, where 9 years later, in 2022, they already accounted for 57% of the total group (181 people). As for the group with moderate CVR, it included 459 people in 2013. Of this pool, 64% had abnormal LDL–c results; 9 years later, the group included 279 participants, and the percentage of those with lipid disorders had risen to 70%. In 2013, the group with high CVR included 361 participants. A total of 91% of participants in this group had elevated LDL–c levels. In 2022, the group size increased to 535 participants, and the percentage of those not meeting the target LDL–c criterion was 92%. There were no individuals in the cohort analyzed who fell into the very high CVR group. Detailed data are presented in Table 1.

### 3.2. Time–Related Change in CVR and How the Selected Effects Modulated It in the Population Sample

**Figure 1:**
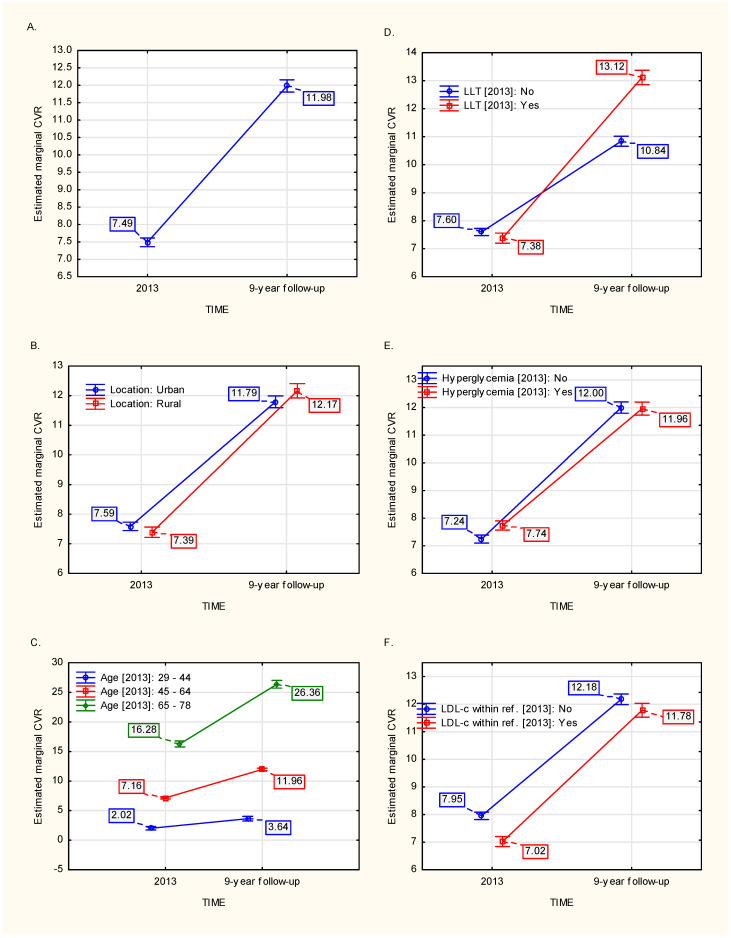
The dynamics of 9–year increase in CVR (**A**) and its change depending on effects (variables): localization (**B**), age (**C**), LLT (**D**), hyperglycemia (**E**) and meeting the LDL–c target for individual CVR (**F**).

Based on the model (Table 2), the whole population showed a steep, approximately 59.94%, increase of the CVR (Figure 1A). Five variables in 2013 had an influence on these 9–year CVD risk dynamics, namely: age (*p* < 0.001), localization (*p* ≈ 0.026), LLT (*p* < 0.001), hyperglycemia (*p* ≈ 0.033), and whether the LDL–c target for the CVR was met (*p* ≈ 0.042).

Individuals living in rural areas were characterized by a 13.81% higher increase in CVR compared to those who lived in urban locations (Figure 1B). Moreover, the 9–year dynamics of CVR were positively associated with age (Figure 1C), reaching over 6.22–fold higher increase in individuals aged 65–78, compared to those aged 29–44. LLT in 2013 (Figure 1D), per se, was associated with approximately 77.16% higher increase of CVR compared to the stratum who was not subject to this treatment. Interestingly, hyperglycemia and meeting the LDL–c target level were associated with: 17.82% lower dynamics of CVR (Figure 1E) and 12.53% higher dynamics of CVR (Figure 1F), respectively.

### 3.3. Factors in 2013: Age, Localization, AT, and Meeting the CVR–Related LDL–c Target Value, Had a Significant Effect on How the LLT Was Associated with the Alteration of the 9–Year CVR Dynamics—Insights from the Analysis of 3rd– and 4th–Degree Interactions

**Figure 2:**
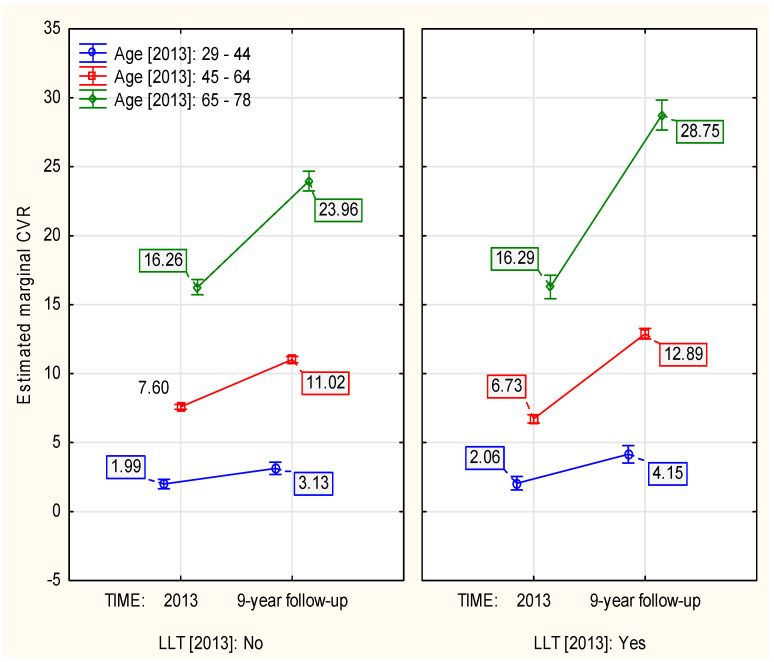
The influence of age in 2013 on the LLT–associated 9–year change in CVR.

Age more steeply influenced the 9–year dynamics of CVR among the individuals administered with LLT in 2013 (*p* ≈ 0.001, Table A1). These individuals, compared to the subjects that did not receive such treatment, showed: 0.95, 2.74, and 3.76 percentage point higher 9–year increase of CVR at age strata: 29–44, 45–64, and 65–78, respectively (Figure 2).

**Figure 3:**
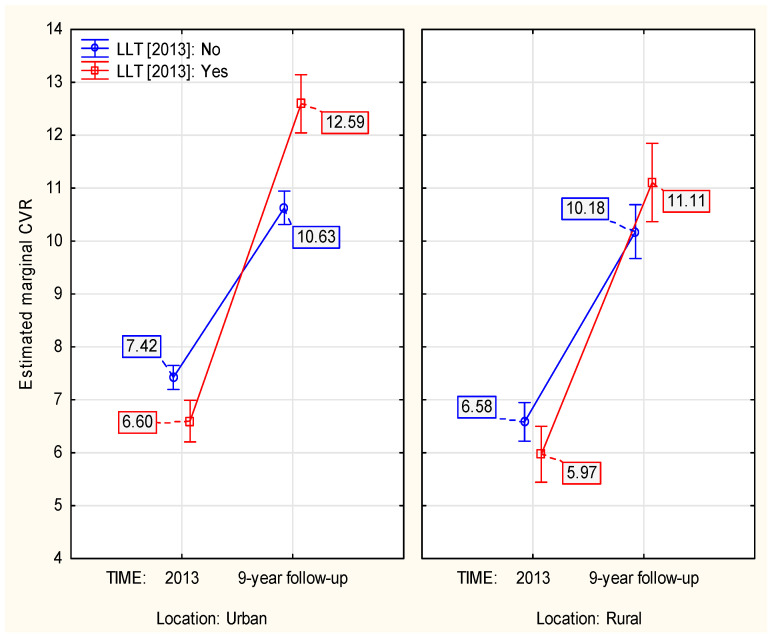
The influence of location in 2013 on the LLT–associated 9–year change in CVR.

The two different localizations (urban/rural) showed different dynamics in how the LLT in 2013 was associated with the 9–year dynamics of CVR (*p* ≈ 0.047, Table A2). The inhabitants of rural areas who were admitted with LLT showed approximately 16.53% lower 9–year increase in CVR compared to individuals living in urban areas. However, among those under no LLT in 2013, the rural areas were associated with 12.15% higher increase in CVR, compared to urban (Figure 3).

**Figure 4:**
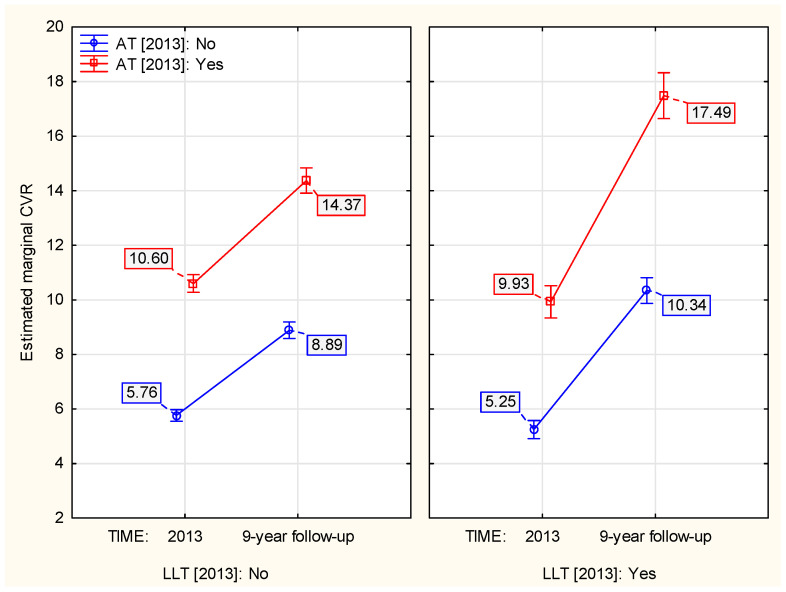
The association between AT in 2013 and the LLT–associated 9–year change in CVR.

The contrast in 9–year CVR dynamics in the context of LLT in 2013 (treatment vs. no treatment) was greater (2.01–fold vs. 1.63, Figure 4) among individuals who received AT in 2013 compared to those who did not receive it (*p* ≈ 0.007, Table A3).

**Figure 5:**
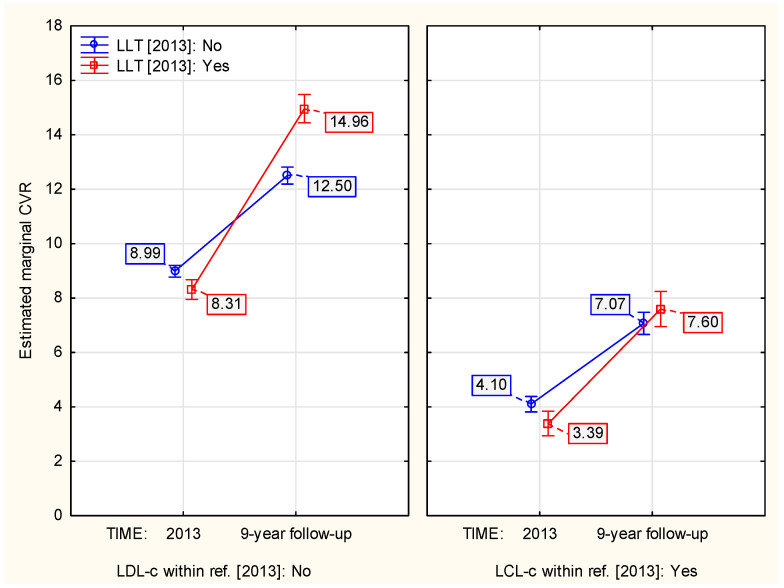
The influence of meeting the target LDL–c level in 2013 on the LLT–associated 9–year change in CVR.

Interestingly, the association between LLT in 2013 and 9–year CVR dynamics was different depending on meeting the target LDL–c levels for CVR in individuals in 2013 (*p* ≈ 0.002, Table A4). The LLT–wise contrast (treatment vs. no treatment) was lower among the individuals who met the target LDL–c level in 2013 compared to those who did not meet it (41.75% difference vs. 89.45% difference, Figure 5).

Since many factors influenced the association between 2013 with LLT and the 9–year dynamics in CVR, higher interactions (between more variables) were analyzed (Tables S1 and S2). Based on them, it could be stated that the association between age and the increase in 9–year CVR (Figure 3) was not affected by either the AT (*p* ≈ 0.885, Table S1) or meeting the LDL–c target in 2013 (*p* ≈ 0.936, Table S2). Moreover, the association between the LLT and the aforementioned risk (Figure 4) was not affected by the differences in localization (*p* ≈ 0.885, Table S1). Interestingly, the simultaneous association between LLT and meeting the LDL–c target (in 2013) and the 9–year dynamics in CVR (Figure 5) was similar in urban and rural areas (*p* ≈ 0.936, Table S2).

## 4. Discussion

Nearly 20 million CVD–related deaths were reported in 2020, an increase of 18.7% from 2010 [12]. This shows the direction of challenges for modern medicine and how important it is to properly manage CVR. Both primary and secondary prevention have a place in the treatment of lipid disorders. In our study, we showed that people with abnormal lipid management results make up a larger proportion of each CVR group, and in subgroups with high CVR, they account for as much as 92%. Despite the passage of more years, greater public awareness, improvements in the diagnosis of hyperlipidemia, and improvements in LLT, the percentage of people with lipid problems has not decreased. Study after study shows how a very low percentage of people who qualify for LLT are actually treated. Mantel–Teeuwisse described the situation in the Dutch population, where only 3.2% of those eligible for LLT were both treated and controlled [13]. Hoerger et al. described in their study that 65% of those eligible for LLT receive no appropriate treatment [14]. Also, we, in our study related to the analysis of the Polish cohort of the PURE study, described that an overwhelming percentage of the group with high and very high CVR had abnormal LDL–c results (91.8% and 98%, respectively). Of these individuals, a significant proportion were not receiving LLT (68.1% and 75%, respectively) [15].

In our study, we showed that patients who used LLT during the initial analysis period had a higher risk of future CVD. This may be related to the phenomenon of reverse causality to which observational epidemiological studies are prone. It describes the relationship between two variables differently than one might expect. In a given situation, one of the variables is the cause, even though it appears to be the effect, and the other, by analogy, is the effect, despite the first impression that it might be the cause. Some refer to reverse causality as “cart–before–the–horse bias” to emphasize the unexpected nature of the correlation. In the study, this phenomenon was observed when the correlation between BMI (body mass index) and mortality was analyzed. Among the elderly, the highest risk was observed at low BMI levels. This was associated with unintentional weight loss with or without a diagnosed disease or a desire to improve health through proper nutrition [16,17].

Similarly, in our considerations, the outcome may have preceded its cause. LLT users, especially in the younger age categories, may have had poorer health, more disease compared to their peers, and thus a higher CVR. Physicians are more likely to start LLT in patients with this profile for secondary prevention than to implement the same treatment for primary prevention in a patient who is asymptomatic and without significant comorbidities. Therefore, it is important that all elements influencing CVR reduction are properly managed. One such component is the use of LLT and the reduction of lipid disorders. Numerous studies show that low adherence to LLT is associated with similar CVD mortality rates as placebo use. In contrast, adherence to LLT significantly reduces [18,19] the rate of cardiovascular events, such as coronary artery disease (CAD) without death, is significantly reduced when pharmacological recommendations are adhered to for a longer time [20]. An analogous situation exists for AT, where it has also been shown that each 20 mmHg reduction in SBP is associated with a twofold reduction in stroke mortality and a twofold difference in the rate of death from vascular causes such as ischemic heart disease [21].

Excessive LDL–c plays an important role in increasing CVR. There are several studies indicating its role in the initiation and progression of vascular disease [22,23]. In the above analysis, it was shown that abnormally elevated LDL–c levels at the beginning of the analysis period were associated with a significantly higher risk of future CVD. Similarly, the difference in future CVR increase between LLT users and non–users was higher in the group of individuals who did not achieve the target LDL–c concentration at the start of the analysis. This is supported by other analyses reporting that lowering LDL–c levels significantly reduces the incidence of myocardial infarction, revascularization or ischemic stroke [24]. Therefore, as Figorilli et al. emphasize, early identification of patients with LDL–c abnormalities and appropriate 10–year CVR stratification are the cornerstones of CVD prevention [25].

Recognition of lipid disorders and implementation of LLT is an essential component of secondary prevention of CVR reduction. The literature consistently shows results indicating a low percentage of patients adhering to recommendations and taking LLT [2,3,4]. Therefore, an important part of patient care is to promote appropriate health–promoting attitudes, including adherence to medical recommendations. To effectively promote the implementation of LLT, it is important to be aware of the limitations that contribute to such a high failure rate of implemented therapy.

Undoubtedly, socioeconomic status has a significant impact. A number of studies have shown the negative impact of rising drug fees on medication adherence [26,27]. LLT is often perceived by patients themselves as a low priority, and taking other medications, such as cardiovascular or antidiabetic drugs, is more important. The phenomenon of polypharmacy, or overuse of pills, also adversely affects the daily routine and every–time adherence. The solution is to use as many compounded preparations as possible. In addition to the excess of pills used daily, non–adherence is also influenced by the multiplicity of chronic diseases and their severity. Latry et al. showed that patients after a recent episode of myocardial infarction or with symptomatic heart disease had a higher rate of adherence to LLT use than those with chronic CAD and minimal symptoms or those using primary prevention [28].

Other reasons for poor adherence to LLT use include inadequate patient knowledge of the role and impact of cholesterol on CVR [29]. Insufficient education at the time of LLT inclusion can lead to a lack of acceptance of lipid disorder disease and skipping appropriate pharmacotherapy in daily life. In the Understanding Statin use in America and Gaps Education (USAGE) study, more than 10,000 respondents confirmed with their answers that adherence to statin use is affected by the manner and comprehensiveness of the physician’s explanation at the time of statin inclusion about the role of cholesterol in CVR and the need to lower it [30]. Communicating potential side effects also has an impact on patient engagement. More extensive explanations of the advantages of statins over side effects, such as muscle symptoms, may contribute to less patient discouragement and more sustained adherence over time [31]. Inadequate information flow may also relate to communication between individual physicians. Kripalani et al. described that hospitalization summaries in the discharge chart are available in less than 34% of cases, and communication between the clinician and the primary care physician occurs in less than 20% of cases [32]. Poor communication can be an obstacle to compliance and discrepancies in medications prescribed in the hospital, which are not then monitored by the primary care physician.

Limitations on the part of the patient, as well as mistakes made by clinicians, affect non–adherence to medical recommendations for LLT. To improve treatment adherence rates, it is important not only to know why LLT is not being used on a daily basis, but also to provide active counseling. It has been shown that nurse–led interventions were associated with better lipid management than standard care. In a study where the intervention arm was a nurse, LDL cholesterol levels < 100 mg/dL were achieved in 97%, compared with 67% for standard care [33]. This shows how important and effective these smallest interventions are. Among these we can include a brief conversation, a reminder to use LLT at each visit. In considering the failure of the LLT used, we should also add the lack of escalation of the dose of the drug used, where low doses are repeatedly maintained.

Our analysis included the Polish cohort of the PURE study, which was designed to compare the lives of urban and rural residents in a number of ways. We showed that rural residents had a more dynamic increase in CVR overall and over time when they did not use LLT at the start of the analysis. In contrast, the increase in CVR over time was lower with hypolipemic treatment at the start of the analysis compared to urban residents. This may be influenced by poorer diagnosis of lipid disorders in rural residents and better treatment since diagnosis. The lifestyle of rural residents may determine higher mortality rates associated with chronic diseases such as cardiovascular disease, respiratory disease, kidney disease and diabetes. Such factors include smoking and obesity. Singh et al. describe that the prevalence of smoking was 16.9% in large metropolitan areas and 26.9% in non–metropolitan areas. Moreover, as recently as the 20th century, lung cancer mortality was significantly higher in metropolitan areas, while it is now higher in non–metropolitan areas [34]. The obesity problem also poses a greater challenge for rural residents. The prevalence of obesity in large metropolitan areas has increased 2.8–fold to 25.9% in recent years, while the prevalence of obesity in non–metropolitan areas has increased 3.5–fold to 33.2% [35]. In addition to individual lifestyle elements influencing the increase in CVR, systemic problems of rural areas must also be taken into account. These include disparities in access to health care. These can stem from cultural and financial constraints, which are often compounded by a shortage of qualified professionals and inadequate public transportation [36].

## 5. Conclusions

Treatment of lipid disorders is an important part of CVD prevention. Previous studies clearly show a low rate of LLT use, even if patients do not reach target LDL–c levels. In our study, we showed that this state of affairs has an impact on future CVR increases. Health–promoting education and raising awareness about the role of proper control and use of LLT is crucial to reducing CVD.

## Figures and Tables

**Table 1:** Distribution of participants by CVR group and meeting the target LDL–c concentration in 2013 and 2022.

	Low CVR	Moderate CVR	High CVR	Very High CVR
2013				
group size	333	459	361	0
have achieved the target LDL–c	229 (69%)	163 (36%)	32 (9%)	
have not achieved the target LDL–c concentration	104 (31%	296 (64%)	329 (91%)	
2022				
group size	181	279	535	0
have achieved the target LDL–c	78 (43%)	84 (30%)	56 (8%)	
have not achieved the target LDL–c concentration	103 (57%)	195 (70%)	(92%)	

**Table 2:** Factorial analysis in context of changes of CVR over the studied time.

Hypothesis	SS	F	*p*
CVR did not change over the studied time…	290.20	41.28	<0.001
… and/or this trend was not affected by age	2149.78	305.83	<0.001
… and/or this trend was not affected by SBP	24.38	3.47	0.063
… and/or this trend was not affected by sex	3.10	0.44	0.507
… and/or this trend was not affected by localization	34.82	4.95	0.026
… and/or this trend was not affected by LLT	663.66	94.41	<0.001
… and/or this trend was not affected by AT	14.29	2.03	0.154
… and/or this trend was not affected by smoking status	10.45	1.49	0.223
… and/or this trend was not affected by hyperglycemic status	31.92	4.54	0.033
… and/or this trend was not affected by dyslipidemia (LDL–c > ref.)	29.23	4.16	0.042
… and/or this trend was not affected by obesity	6.99	0.99	0.319
–	7633.75		

Abbreviations: AT, anti–hypotensive treatment; LDL–c, low–density lipoprotein cholesterol; LLT, lipid–lowering therapy; SBP, systolic blood pressure; SS, sum of squares.

## Data Availability

The data are available upon request.

## Supplementary Materials

The following supporting information can be downloaded at: https://www.mdpi.com/article/10.3390/jcm13133728/s1, Table S1: Three–way modulation of the change of CVR over time by: localization, LLT and AT—insights from logistic regression model with interactions. Table S2: Three–way modulation of the change of CVR over time by: localization, LLT and having LDL–c levels within the reference values in 2013—insights from logistic regression model with interactions.

## Appendix A

**Table A1:** The influence of age in 2013 on the LLT–associated 9–year change in CVR—tabularized summary of the used logistic regression model.

Effect/Interaction	Hypothesis	SS	df	MS	F	*p*
TIME	CVR did not change over the studied time…	6251.61	1	6251.61	844.55	<0.001
(A) TIME × LLT [2013]	… and/or this trend was not affected by LLT	410.44	1	410.44	55.45	<0.001
(B) TIME × Age [2013]	… and/or this trend was not affected by age	1868.99	2	934.49	126.24	<0.001
TIME × LLT [2013] × Age [2013]	… and both above statements are not affected by differences in: age (A) or LLT (B)	103.52	2	51.76	6.99	0.001
Error		7898.24	1067	7.40		

SS—sums of squares; df—degrees of freedom; MS—mean square.

**Table A2:** The influence of location in 2013 on the LLT–associated 9–year change in CVR—tabularized summary of the used logistic regression model.

Effect/Interaction	Hypothesis	SS	df	MS	F	*p*
TIME	CVR did not change over the studied time…	7509.97	1	7509.97	820.27	<0.001
(A) TIME × Localization	… and/or this trend was not affected by localization	5.15	1	5.15	0.56	0.454
(B) TIME × LLT [2013]	… and/or this trend was not affected by LLT	437.21	1	437.21	47.75	<0.001
TIME × Localization ×LLT [2013]	… and both above statements were not affected by differences in: LLT (A) or localization (B)	36.37	1	36.37	3.97	0.047
Error		9787.17	1069	9.16		

SS—sums of squares; MS—mean square; df—degrees of freedom.

**Table A3:** The association between AT in 2013 and the LLT–associated 9–year change in CVR—tabularized summary of the used logistic regression model.

Effect/Interaction	Hypothesis	SS	df	MS	F	*p*
TIME	CVR did not change over the studied time…	7653.22	1	7653.22	849.73	<0.001
(A) TIME × LLT [2013]	… and/or this trend was not affected by LLT	663.04	1	663.04	73.62	<0.001
(B) TIME × AT [2013]	… and/or this trend was not affected by AT	194.08	1	194.08	21.55	<0.001
TIME × LLT [2013] × HT [2013]	… and both above statements were not affected by differences in: AT (A) or LLT (B)	65.59	1	65.59	7.28	0.007
Error		9628.09	1069	9.01		

df—degrees of freedom; MS—mean square; SS—sums of squares.

**Table A4:** The influence of meeting the target LDL–c level in 2013 on the LLT–associated 9–year change in CVR—tabularized summary of the used logistic regression model.

Effect/Interaction	Hypothesis	SS	df	MS	F	*p*
TIME	CVR did not change over the studied time…	7540.81	1	7540.81	840.51	<0.001
(A) TIME × LLT [2013]	… and/or this trend was not affected by LLT	479.22	1	479.22	53.42	<0.001
(B) TIME × LDL–c within reference value [2013]	… and/or this trend was not affected by dyslipidemia	223.45	1	223.45	24.91	<0.001
TIME × LLT [2013] × LDL–c within reference value [2013]	… and both above statements were not affected by differences in: dyslipidemic status (A) or LLT (B)	89.43	1	89.43	9.97	0.002
Error		9590.71	1069	8.97		

MS—mean square; df—degrees of freedom; SS—sums of squares.

## References

[1] Di Cesare M., Bixby H., Gaziano T., Hadeed L., Kabudula C., Vaca McGhie D., Mwangi J., Pervan B., Perel P., Piñeiro D. (2023). World Heart Report 2023: Confronting the World’s Number One Killer.

[2] National Health Fund of Poland (2022). RAPORT NFZ: Nadciśnienie Tętnicze, Poland Government. https://www.nfz.gov.pl/aktualnosci/aktualnosci-centrali/raport-nfz-nadcisnienie-tetnicze,7352.html.

[3] Pająk A., Szafraniec K., Polak M., Polakowska M., Kozela M., Piotrowski W., Kwaśniewska M., Podolecka E., Kozakiewicz K., Tykarski A. (2016). Changes in the prevalence, treatment, and control of hypercholesterolemia and other dyslipidemias over 10 years in Poland: The WOBASZ study. Pol. Arch. Med. Wewn..

[4] Nowicki G., Ślusarska B., Brzezicka A. (2009). Analiza stanu wiedzy o czynnikach ryzyka chorób układu sercowo-naczyniowego wśród osób pracujących. Nurs. Top..

[5] Visseren F.L.J., Mach F., Smulders Y.M., Carballo D., Koskinas K.C., Bäck M., Benetos A., Biffi A., Boavida J.M., Capodanno D. (2021). 2021 ESC guidelines on cardiovascular disease prevention in clinical practice. Eur. Heart J..

[6] Livingstone S.J., Looker H.C., Hothersall E.J., Wild S.H., Lindsay R.S., Chalmers J., Cleland S., Leese G.P., McKnight J., Morris A.D. (2012). Risk of cardiovascular disease and total mortality in adults with type 1 diabetes: Scottish registry linkage study. PLoS Med..

[7] Teo K., Chow C.K., Vaz M., Rangarajan S., Yusuf S., PURE Investigators-Writing Group (2009). The Prospective Urban Rural Epidemiology (PURE) study: Examining the impact of societal influences on chronic noncommunicable diseases in low-, middle-, and high-income countries. Am. Heart J..

[8] Zatońska K., Zatoński W.A., Szuba A. (2016). Prospective urban and rural epidemiology Poland—Study design. J. Health Inequal..

[9] World Health Organization Disease Burden and Mortality Estimates. https://www.who.int/data/gho/data/themes/mortality-and-global-health-estimates.

[10] SCORE2 Working Group, ESC Cardiovascular Risk Collaboration (2021). SCORE2 risk prediction algorithms: New models to estimate 10-year risk of cardiovascular disease in Europe. Eur. Heart J..

[11] SCORE2-OP Working Group, ESC Cardiovascular Risk Collaboration (2021). SCORE2-OP risk prediction algorithms: Estimating incident cardiovascular event risk in older persons in four geographical risk regions. Eur. Heart J..

[12] Tsao C.W., Aday A.W., Almarzooq Z.I., Alonso A., Beaton A.Z., Bittencourt M.S., Boehme A.K., Buxton A.E., Carson A.P., Commodore-Mensah Y. (2022). Heart Disease and Stroke Statistics-2022 Update: A Report From the American Heart Association. Circulation.

[13] Mantel-Teeuwisse A.K., Verschuren W.M., Klungel O.H., Kromhout D., Lindemans A.D., Avorn J., Porsius A.J., de Boer A. (2003). Undertreatment of hypercholesterolaemia: A population-based study. Br. J. Clin. Pharmacol..

[14] Hoerger T.J., Bala M.V., Bray J.W., Wilcosky T.C., LaRosa J. (1998). Treatment patterns and distribution of low-density lipoprotein cholesterol levels in treatment-eligible United States adults. Am. J. Cardiol..

[15] Lubieniecki P., Wołyniec M., Połtyn-Zaradna K., Zatońska K., Szuba A. (2024). Lipid-Lowering Therapy in PURE Poland Cohort Study. J. Clin. Med..

[16] Heiat A., Vaccarino V., Krumholz H.M. (2001). An evidence-based assessment of federal guidelines for overweight and obesity as they apply to elderly persons. Arch. Intern. Med..

[17] Besser L.M., Brenowitz W.D., Meyer O.L., Hoermann S., Renne J. (2021). Methods to Address Self-Selection and Reverse Causation in Studies of Neighborhood Environments and Brain Health. Int. J. Environ. Res. Public Health.

[18] Perreault S., Dragomir A., Blais L., Bérard A., Lalonde L., White M., Pilon D. (2009). Impact of better adherence to statin agents in the primary prevention of coronary artery disease. Eur. J. Clin. Pharmacol..

[19] Ho P.M., Spertus J.A., Masoudi F.A., Reid K.J., Peterson E.D., Magid D.J., Krumholz H.M., Rumsfeld J.S. (2006). Impact of medication therapy discontinuation on mortality after myocardial infarction. Arch. Intern. Med..

[20] Bouchard M.H., Dragomir A., Blais L., Bérard A., Pilon D., Perreault S. (2007). Impact of adherence to statins on coronary artery disease in primary prevention. Br. J. Clin. Pharmacol..

[21] Lewington S., Clarke R., Qizilbash N., Peto R., Collins R., Prospective Studies Collaboration (2002). Age-specific relevance of usual blood pressure to vascular mortality: A meta-analysis of individual data for one million adults in 61 prospective studies. Lancet.

[22] Tabas I., Williams K.J., Borén J. (2007). Subendothelial lipoprotein retention as the initiating process in atherosclerosis: Update and therapeutic implications. Circulation.

[23] Ference B.A., Ginsberg H.N., Graham I., Ray K.K., Packard C.J., Bruckert E., Hegele R.A., Krauss R.M., Raal F.J., Schunkert H. (2017). Low-density lipoproteins cause atherosclerotic cardiovascular disease. 1. Evidence from genetic, epidemiologic, and clinical studies. A consensus statement from the European Atherosclerosis Society Consensus Panel. Eur. Heart J..

[24] Baigent C., Blackwell L., Emberson J., Holland L.E., Reith C., Bhala N., Peto R., Barnes E.H., Keech A., Cholesterol Treatment Trialists’ (CTT) Collaboration (2010). Efficacy and safety of more intensive lowering of LDL cholesterol: A meta-analysis of data from 170,000 participants in 26 randomised trials. Lancet.

[25] Figorilli F., Mannarino M.R., Bianconi V., Pirro M. (2022). Cholesterol-Lowering Therapy in Patients at Low-to-Moderate Cardiovascular Risk. High Blood Press. Cardiovasc. Prev..

[26] Simoens S., Sinnaeve P.R. (2014). Patient co-payment and adherence to statins: A review and case studies. Cardiovasc. Drugs Ther..

[27] Choudhry N.K., Avorn J., Glynn R.J., Antman E.M., Schneeweiss S., Toscano M., Reisman L., Fernandes J., Spettell C., Lee J.L. (2011). Full coverage for preventive medications after myocardial infarction. N. Engl. J. Med..

[28] Latry P., Molimard M., Dedieu B., Couffinhal T., Bégaud B., Martin-Latry K. (2011). Adherence with statins in a real-life setting is better when associated cardiovascular risk factors increase: A cohort study. BMC Cardiovasc. Disord..

[29] Gazmararian J.A., Kripalani S., Miller M.J., Echt K.V., Ren J., Rask K. (2006). Factors associated with medication refill adherence in cardiovascular-related diseases: A focus on health literacy. J. Gen. Intern. Med..

[30] Cohen J.D., Brinton E.A., Ito M.K., Jacobson T.A. (2012). Understanding Statin Use in America and Gaps in Patient Education (USAGE): An internet-based survey of 10,138 current and former statin users. J. Clin. Lipidol..

[31] Wei M.Y., Ito M.K., Cohen J.D., Brinton E.A., Jacobson T.A. (2013). Predictors of statin adherence, switching, and discontinuation in the USAGE survey: Understanding the use of statins in America and gaps in patient education. J. Clin. Lipidol..

[32] Kripalani S., LeFevre F., Phillips C.O., Williams M.V., Basaviah P., Baker D.W. (2007). Deficits in communication and information transfer between hospital-based and primary care physicians: Implications for patient safety and continuity of care. JAMA.

[33] Ruiz-Bustillo S., Ivern C., Badosa N., Farre N., Marco E., Bruguera J., Cladellas M., Enjuanes C., Cainzos-Achirica M., Marti-Almor J. (2019). Efficacy of a nurse-led lipid-lowering secondary prevention intervention in patients hospitalized for ischemic heart disease: A pilot randomized controlled trial. Eur. J. Cardiovasc. Nurs..

[34] Singh G.K., Siahpush M., Williams S.D. (2012). Changing urbanization patterns in US lung cancer mortality, 1950–2007. J. Community Health.

[35] Schiller J.S., Lucas J.W., Ward B.W., Peregoy J.A. (2012). Summary health statistics for U.S. adults: National Health Interview Survey, 2010. Vital Health Stat..

[36] Douthit N., Kiv S., Dwolatzky T., Biswas S. (2015). Exposing some important barriers to health care access in the rural USA. Public Health.

