# Supplementary material for: The Dynamics of Cardiovascular Risk—An Analysis of the Prospective Urban Rural Epidemiology (PURE) Poland Cohort Study
Source: J Clin Med. 2024 Jun 26;13(13):3728. doi: 10.3390/jcm13133728 (PMC11242048; doi:10.3390/jcm13133728)
Supplement: Supplementary file 1 [file jcm-13-03728-s001.zip › jcm-3033220-supplementary.pdf]

Supplementary Materials

Table S1. Three-way modulation of the change of CVR over time by: localization, LLT and AT – insights from logistic regression model with interactions

**Effect/interaction**	**Hypothesis**	**SS**	**df**	**MS**	**F**	**p**
TIME	CVR did not change over the studied time…	6968,474	1	6968,474	774,9417	0,000000
A) TIME*Localization	… and/or this trend was not affected by localization	11,564	1	11,564	1,2860	0,257050
B) TIME*LLT [2013]	… and/or this trend was not affected by LLT	525,216	1	525,216	58,4076	0,000000
C) TIME*AT [2013]	… and/or this trend was not affected by AT	188,313	1	188,313	20,9416	0,000005
D) TIME*Localization*LLT [2013]	… and statements A and B were not affected by differences in: LLT (A) or localization (B)	42,877	1	42,877	4,7682	0,029208
E) TIME*Localization*AT [2013]	… and statements A and C were not affected by differences in: AT (A) or localization (B)	0,463	1	0,463	0,0515	0,820460
F) TIME*LLT [2013]*AT [2013]	… and statements B and C were not affected by differences in: AT (B) or LLT (C)	68,741	1	68,741	7,6445	0,005793
TIME*LLT [2013]*AT [2013]*Localization	… and statements D, E and F were not affected by: localization (D, E), LLT (D, F) or AT (E, F)	0,188	1	0,188	0,0209	0,884974
Error		9576,753	1065	8,992		

SS - sums of squares; df - degrees of freedom; MS - mean square

Table S2. Three-way modulation of the change of CVR over time by: localization, LLT and having LDL-c levels within the reference values in 2013 – insights from logistic regression model with interactions

**Effect/interaction**	**Hypothesis**	**SS**	**df**	**MS**	**F**	**p**
TIME	CVR did not change over the studied time…	6540,113	1	6540,113	729,2251	0,000000
A) TIME*Localization	… and/or this trend was not affected by localization	7,143	1	7,143	0,7964	0,372370
B) TIME*LLT [2013]	… and/or this trend was not affected by LLT	343,987	1	343,987	38,3547	0,000000
C) TIME*LDL-c within ref. [2013]	… and/or this trend was not affected by dyslipidemic status	211,317	1	211,317	23,5619	0,000001
D) TIME*Localization*LLT [2013]	… and statements A and B were not affected by differences in: LLT (A) or localization (B)	35,095	1	35,095	3,9131	0,048169
E) TIME*Localization*LDL-c within ref. [2013]	… and statements A and C were not affected by differences in: dyslipidemic status (A) or localization (B)	2,070	1	2,070	0,2308	0,631018
F) TIME*LLT [2013]*LDL-c within ref. [2013]	… and statements B and C were not affected by differences in: dyslipidemic status (B) or LLT (C)	79,075	1	79,075	8,8169	0,003051
TIME*LLT [2013]*LDL-c within ref. [2013]*Localization	… and statements D, E and F were not affected by: localization (D, E), LLT (D, F) or dyslipidemic status (E, F)	0,058	1	0,058	0,0064	0,936099
Error		9551,536	1065	8,969		

SS - sums of squares; df - degrees of freedom; MS - mean square
